# Rheumatoid arthritis and incident fracture in women: a case–control study

**DOI:** 10.1186/1471-2474-15-13

**Published:** 2014-01-09

**Authors:** Sharon L Brennan, Liesje Toomey, Mark A Kotowicz, Margaret J Henry, Hedley Griffiths, Julie A Pasco

**Affiliations:** 1 School of Medicine, Deakin University, Geelong, Victoria, Australia; 2 NorthWest Academic Centre, The University of Melbourne, St Albans, Victoria, Australia; 3 Australian Institute for Musculoskeletal Science (AIMSS), The University of Melbourne, St Albans, Victoria, Australia; 4 Barwon Rheumatology Service, Geelong, Victoria, Australia

**Keywords:** Incident fracture, Rheumatoid arthritis, Epidemiology, Women

## Abstract

**Background:**

To examine fracture incidence in women with rheumatoid arthritis (RA) for an entire geographical region of south-eastern Australia.

**Methods:**

Women aged 35 years and older, resident in the Barwon Statistical Division (BSD) and clinically diagnosed with RA 1994–2001 were eligible for inclusion as cases (n = 1,008). The control population (n = 172,422) comprised the entire female BSD population aged 35 years and older, excluding those individuals identified as cases. Incident fractures were extracted from the prospective Geelong Osteoporosis Study Fracture Grid. We calculated rate ratios (RR) and 95% confidence intervals (CI) to compare the age-adjusted rate of fracture between the RA and non-RA populations, and used a chi-square test to compare proportions of fractures between women with and without RA, and a two-sided Mann–Whitney U-test to examine age-differences.

**Results:**

Among 1,008 women with RA, 19 (1.9%) sustained a fracture, compared to 1,981 fractures sustained by the 172,422 women without RA (1.2%). Fracture rates showed a trend for being greater among women diagnosed with RA (age-adjusted RR 1.43, 95%CI 0.98-2.09, *p* = 0.08). Women with RA sustained vertebral fractures at twice the expected frequency, whereas hip fractures were underrepresented in the RA population (*p* < 0.001). RA status was not associated with the likelihood of sustaining a fracture at sites adjacent to joints most commonly affected by RA (*p* = 0.22).

**Conclusion:**

Given that women with RA have a greater risk of fracture compared to women without RA, these patients may be a suitable target population for anti-resorptive agents; however, larger studies are warranted.

## Significance and innovation

Previous investigations regarding fracture risk in women with rheumatoid arthritis (RA) have been confined to specific sub-populations or a limited number of fracture sites: there is currently little known regarding the overall risk of fracture associated with RA.

Women with RA have an increased risk of fracture compared to women without RA.

Fractures are more likely to occur at major osteoporotic sites than at sites adjacent to the joints affected by RA.

Women with RA may be a suitable target population for anti-resorptive agents to reduce fracture risk.

## Background

Rheumatoid arthritis (RA) is an autoimmune disease characterised by symmetric joint inflammation, stiffness and pain, and by muscular weakness around the affected joints. RA is the most severe form of arthritis [1]. The worldwide prevalence of RA is approximately 1%, and the most prevalent autoimmune condition in Australia, affecting 2.1% of the population, 63% of whom are women [1]. The onset of RA generally occurs between 40–60 years of age, peaking in females aged 65–74 years [1].

Drug therapies in RA are aimed at maintaining or improving functional status, and the more commonly prescribed anti-inflammatory drugs in the treatment of RA include non-steroidal anti-inflammatory drugs (NSAIDs) or glucocorticoid therapy; the latter used in cases of active and severely disabling RA [2]. A well-documented effect of glucocorticoids is that they disrupt bone metabolism, causing an increase in bone resorption and a decrease in formation via several different mechanisms [2]. However, glucocorticoids are also potent immunosuppressants and can therefore reduce bone damage associated with disease activity in RA [3,4].

Bone loss in RA can also occur independently of corticosteroid therapy, and the localised erosion of bone around disease joints is a characteristic of RA [5]. Whilst osteoclastogenesis is induced in the RA inflammatory response [6,7] resulting in bone resorption, some erosions of bone appear to be physically rather than chemically mediated [8]. Generalised skeletal bone loss is also associated with RA. Studies have shown that bone mineral density (BMD) at the hip and spine is lower in RA patients compared to the general population [6,9,10]. It is plausible that a number of different factors associated with RA may be involved in the process of generalised bone loss. Disease activity and duration, and corticosteroid use have been implicated in bone loss at both the hip and spine whereas studies suggest decreased physical activity may be only associated with decreased hip BMD [3,6,10,11].

The presence of these factors, in combination with older age, suggests that RA patients may be a population at increased risk of fracture; an association reported by some studies [12-14]. However, given that many previous investigations have been confined to specific sub-populations, small sample sizes, or a limited number of fracture sites, there is currently little known regarding the overall risk of fracture associated with RA. We aimed to examine; (a) whether fracture incidence would differ between women with RA and women without RA, and (b) whether the site-specific fracture would differ between women with and without RA, using an entire statistical division of south-eastern Australia as the study population.

## Methods

### Rheumatoid arthritis (cases)

Women aged 35 years and older, resident in the Barwon Statistical Division (BSD), and with RA diagnosis between 1994 and 2001 were eligible for inclusion in these analyses. RA status was ascertained by manually checking the eligibility criteria (age, sex, and resident status) against records of the Barwon Rheumatology Service, the region’s sole rheumatology practice that was established in the early 1990s. RA was diagnosed by the treating rheumatologists at the Barwon Rheumatology Service, Victoria, Australia, using the predominant diagnostic set of criteria published by the American Rheumatism Association in 1988 [5].

### Non-RA population (controls)

The control population comprised the entire BSD population aged 35 years and older, excluding those individuals identified in the RA population, in order to provide two mutually exclusive groups for case–control analyses. The 1996 Australian Bureau of Statistics (ABS) Census count for the BSD female population aged 35 years and older was 57,454 [15]. Control population figures for the years of 1994 and 1995 were extrapolated using the 1996 population figures.

### Ascertainment of incident fracture

The Geelong Osteoporosis Study (GOS) Fracture Grid (GOS-FracGrid) prospectively documents all incident fractures sustained by residents of the BSD using a validated method to identify fractures from original radiological reports, as previously published [16]. The first occurring incident fracture for all women in the BSD aged 35 years and older was identified for a two year period 1994–96 [15]. To calculate the RA population fracture incidence rate in person-years, fractures occurring in the RA population 1994–1996 were used, in order to examine whether the fracture incidence in women with RA was increased compared to women without RA. The GOS-FracGrid uses the International Classification of Diseases, Ninth Revision (ICD-9) [17] to code the site of fracture, as previously published. In order to increase the sample size to examine site-specific fractures, we included every fracture case that occurred from 1994–2001 for analyses, although where multiple fractures, only the first fracture at any individual site was included.

Approval for this study was provided by the Barwon Health Human Research and Ethics Committee (HREC). For the RA cases, written informed consent to access fracture records was provided by living patients, and Barwon Health HREC provided ethical approval to access the electronic medical records of patients with RA that were deceased. Development of the comprehensive GOS-FracGrid was approved via a waiver of consent provided by Barwon Health HREC.

### Statistical analyses

Age- and sex-specific, total-fracture and site-specific fracture incidence rates in person years for the entire BSD population have been published using the data collected by GOS 1994–1996 [18]; the corresponding rates for the non-RA population included in our current study were derived from those data by removing the individuals with RA. We made the assumption that fracture rates remained constant between 1994–2001 and this assumption was used to calculate the expected fracture numbers in the RA cases.

Age-adjusted rate ratios (RR) and 95% confidence intervals (CI) were calculated to compare the rate of incident fracture between women with and without RA. When calculating the age-adjusted RR, the following categorical age groupings were used; 35–54 years, 55–74 years, and 75+ years, so as to ensure that each age stratum held an appropriate number of fractures.

To examine site-specific fracture and RA, we used a chi-square test to examine differences in proportions of fractures between the two groups, and a two-sided Mann–Whitney U-test was used to examine age-differences.

All analyses were performed using Minitab (Version 12.0; Minitab, State College, PA).

## Results

Mean age for RA cases was 64 years (IQR 52–71) and 53 years (IQR 43–68) for the non-RA population. A Mann–Whitney test revealed that the median age for RA cases was older than that of the control population (T = 9.2, *p* < 0.001). At the onset of RA, mean (± SD) age was 53 ± 16 years, and the year of RA onset ranged between 1940 and 2001, with the majority of RA patients developing the disease post-1989. The prevalence of RA in women was 0.6%.

### Fracture incidence

Table 1 presents the number and percentage of fractures according to skeletal site, in women with and without RA. Table 2 presents the numbers of fractures sustained by women with and without RA for the study period of 1994–1996 in the BSD, stratified by year of data ascertainment. Of the 1,008 women with RA, 19 (1.9%) sustained a fracture compared to 1,981 fractures sustained by the 172,422 women without RA (1.2%).

**Table 1:** Number and percentage of fractures by skeletal site among women with and without RA

**Skeletal site**	**Rheumatoid arthritis (cases)**	**Non-rheumatoid arthritis (controls)**
Face	1 (1.2%)	13 (0.8%)
Skull	0	2 (0.1%)
Vertebra	26 (31.7%)	243 (15.6%).
Rib	4 (4.9%)	64 (4.1%)
Pelvis	2 (2.4%)	63 (4.0%)
Clavical	0	14 (0.9%)
Scapula	0	7 (0.5%)
Humerus	12 (14.6%)	128 (8.2%)
Forearm	3 (3.7%)	108 (6.9%)
Colles’	6 (7.3%)	211 (13.5%)
Carpal bone	1 (1.2%)	18 (1.2%)
Hand	1 (1.2%)	23 (1.5%)
Finger	1 (1.2%)	36 (2.3%)
Hip	6 (7.3%)	321 (20.5%)
Upper leg	4 (4.9%)	25 (1.6%)
Patella	4 (4.9%)	21 (1.3%)
Lower leg	3 (3.7%)	85 (5.4%)
Ankle	4 (4.9%)	100 (6.4%)
Foot	3 (3.7%)	55 (3.5%)
Toe	1 (1.2%)	26 (1.7%)
**Total**	82 (100%)	1563 (100%)

**Table 2:** Numbers of fractures in women with and without RA (1994–96) in the Barwon Statistical Division, stratified by year of data ascertainment

	**Rheumatoid arthritis (cases)**		**Non-rheumatoid arthritis (controls)**	
***Year***	***RA population at risk***	***Fracture numbers (%)***	Population at risk*	Fracture numbers (%)**
1994	319	3 (0.94%)	57,491	601 (1.04%)
1995	333	5 (1.50%)	57,477	690 (1.20%)
1996	356	11 (3.09%)	57,454	690 (1.20%)
Total	1,008	19 (1.88%)	172,422	1,981 (1.15%)

*Control population figures were extrapolated using 1996 Census data [15].

**Raw fractures figures were ascertained for 1994–1996 from Sanders et al. [18].

Crude fracture incidence rates for women with and without RA were 188 per 10,000 person years and 114 per 10,000 person years, respectively. In age-adjusted analyses, a trend was observed for women with RA to be 43% more likely to fracture than non-RA women of the same age (age-adjusted RR 1.43, 95%CI 0.98, 2.09, *p* = 0.08).

### Site-specific fracture

In women with RA, 82 fractures were sustained during the study period, whereas 1,563 fractures were sustained by the non-RA group (Table 3). No age-differences at time of fracture were observed between women with RA or in women without RA (72 *vs*.74 years, respectively, U-1.00, 95%CI −2.00, 4.00, *p* = 0.38).

**Table 3:** Observed and expected number of fractures at primary osteoporotic sites, and sites adjacent to the joints most commonly affected early in RA in the Barwon Statistical Division

	**Rheumatoid arthritis (cases)**		**Non-rheumatoid arthritis (controls)**		**Chi-square test**	
	**Observed**	**Expected**	**Observed**	**Expected**	**χ**^**2**^**value**	**p value**
*Primary osteoporotic sites*						
Vertebral	26	13.4	243	255.6	21.71	<0.001
Colles’	6	10.8	211	206.2		
Hip	6	16.3	321	310.7		
Other	44	41.5	788	790.5		
*Sites adjacent to joints most commonly affected early in RA*						
Colles’/Carpal/Hand/Fingers	9	14.8	288	282.2	3.02	0.22
Foot/Toes	4	4.2	81	80.8		
Other	69	63.0	1,194	1200		

The proportions of classic ‘osteoporotic’ fractures (Colles’, vertebral, and hip) sustained by the RA and non-RA groups differed significantly (χ^2^ = 21.71, *p* < 0.001). The greatest difference between observed and expected values occurred for the vertebral and hip fractures, whereby women with RA sustained vertebral fractures at twice the expected frequency (26.0% versus 13.4), whereas hip fractures were underrepresented in the RA population (6% versus 16.3) (χ^2^ = 21.71, *p* = <0.001). No difference was observed between the RA and non-RA groups in the likelihood of sustaining a fracture at sites adjacent to the joints most commonly affected in RA (χ^2^ = 3.02, *p* = 0.22).

## Discussion

Our data show a greater risk of fracture for women with RA compared to non-RA women, with no age difference at the time of fracture regardless of RA status. Fractures are more likely to occur at major osteoporotic sites such as vertebral, hip or Colles’, than at sites adjacent to the joints affected by RA.

The sites of hand, fingers, feet and toes, are joints most commonly affected in RA, and therefore the regions more likely to have suffered localized bone erosion resulting in low BMD. In the general female population, fractures at these sites most commonly occur in females aged less than 50 years [18,19]. It is plausible that in females with RA, the most influential risk factors for fracture are the same as those factors which predispose non-RA women to fracture, for instance menopausal status and age, rather than disease specific factors such as RA disease activity or duration; a suggestion supported by others [13,20]. We may also speculate that localized bone erosion around joints affected by RA has less influence of the risk of fracture compared to the reduced BMD seen at the hip and spine in RA patients.

In our study, women with RA sustained vertebral fractures at twice the expected frequency; consistent with other studies that examined associations between vertebral fractures and RA [14,21,22]. The most plausible explanation for this is the increased rate of detection of vertebral fractures, rather than a true increase in incidence. It has been estimated that approximately two thirds of vertebral fractures are asymptomatic [23,24], resulting in many vertebral fractures in the general population remaining undiagnosed. In contrast, RA patients have overall poorer health [25,26], with a consequent increase in the likelihood for incidental detection of asymptomatic vertebral fractures. However, in a case–control study of Norwegian females, those with RA were more likely to have at least one vertebral deformity compared to those without RA (22% *vs.* 15%), and significantly more likely to have multiple vertebral deformities (11% *vs.* 5%, respectively). The presence of deformities is important, given they have been associated with subsequent fracture [14,27]. Importantly, our data are in the majority consistent with studies from other countries, including Finland [28], Norway [29], the UK [23] and the US [13]. Taken in context, our observations suggest that RA patients may be a suitable target population for anti-resorptive agents.

However, we observed a lower than expected rate of hip fracture in our RA population. This observation may be explained by the unmeasured severity of disease; more severe RA disease is known to increase the likelihood of a sedentary lifestyle, which might negatively influence lean mass and muscle strength [30] and increase falls risk, with a subsequent increase in fracture risk. Indeed, it has been reported that a fear of falling is common in RA populations [31]. Alternatively, and given that we captured all RA patients, some of whom may be early in the disease pathogenesis, it is likely that hip fracture may be associated with later or long standing disease [21], a greater severity of RA disease [32], a prolonged use of medications associated with RA, or other unmeasured factors. We did not ascertain reasons for fracture in the RA or non-RA population; however, incident hip fractures in our controls may plausibly be related to increased risk taking behavior, or in the very least, less sedentary behavior when compared to the RA population. Whilst an increased hip fracture incidence is often reported in RA populations compared to non-RA populations, some studies have acknowledged that their RA sample included a higher proportion of osteoporotic patients, represented by low bone mineral density, compared to the general population [14]; ours was a comprehensive study of an entire region.

Our study has some strength. We derived our non-RA population at risk from official 1996 Census counts of the ABS [15]. By use of the GOS-FracGrid, which documents incident fractures for the entire BSD region ascertained from radiological reports, we were therefore able to directly calculate the fracture incidence rates for the RA and non-RA populations. Whilst other studies have examined associations between RA and fractures at various sites, a unique feature of our work is that our fracture ascertainment methodology enabled us to identify all incident fractures across an entire regional area and at all skeletal sites. Furthermore the fractures investigated in our study were not self-reported, nor did they require hospital admission (as we were not reliant on hospital admission and/or discharge data). Our study also has some limitations. Whereas our validated method of identifying fractures from original radiological reports tested the possibility of false-positives, we cannot exclude the possibility that false-negatives (failure to report a fracture) may exist. False-negatives in radiology reporting would likely result in an underestimation of the association between fracture incidence and RA; for instance not all fractures of the fingers or toes may undergo radiography. Our identification of RA patients would exclude those residents of the BSD who may have chosen to visit rheumatologists outside the region for ongoing care; however, rheumatologists practicing at the Barwon Rheumatology Service suggest that the number of RA sufferers in the region whom they had not seen for at least one consultation would likely be negligible. It is plausible that we may have missed fractures that occurred outside the BSD; a factor that may have influenced our lower than expected rate of hip fractures in the RA population. Given our sample size, and the wide age strata necessarily employed for analyses, we cannot exclude that residual confounding may influence our results. Finally, we were unable to account for medication use, the duration of RA, or co-morbidities, which may influence the observed associations. Although it has been suggested that glucocorticoid use may not confound the association between fracture and RA [23], indeed glucocorticoid use has been documented as both negatively and positively influencing bone damage.

## Conclusion

In conclusion, our data indicate that women with RA have an increased risk of fracture compared to women without RA. Furthermore, fractures are more likely to occur at major osteoporotic sites such as vertebral, hip or Colles’, than at sites adjacent to the joints affected by RA. In light of these data, we suggest that RA patients may be a suitable target population for anti-resorptive agents such as bisphosphonates, however we suggest that larger studies are warranted.

## Competing interests

SLB, LT, MAK, MJH, and JAP declare that they have no competing interests. HG is a lead Rheumatologist at the Barwon Rheumatology Service.

## Authors’ contributions

SLB drafted the manuscript. LT performed the statistical analysis, and MAK, MJH, HG, and JAP supervised the analysis. All authors interpreted the data, and guided and reviewed the manuscript. All authors read and approved the final manuscript.

## Pre-publication history

The pre-publication history for this paper can be accessed here:

http://www.biomedcentral.com/1471-2474/15/13/prepub
